# Plasma and Peritoneal Fluid ZEB Levels in Patients with Endometriosis and Infertility

**DOI:** 10.3390/biomedicines10102460

**Published:** 2022-10-01

**Authors:** Paweł Bartnik, Joanna Kacperczyk-Bartnik, Ksawery Goławski, Janusz Sierdziński, Grzegorz Mańka, Mariusz Kiecka, Michał Lipa, Damian Warzecha, Robert Spaczyński, Piotr Piekarski, Beata Banaszewska, Artur J. Jakimiuk, Tadeusz Issat, Wojciech Rokita, Jakub Młodawski, Maria Szubert, Piotr Sieroszewski, Grzegorz Raba, Kamil Szczupak, Tomasz Kluz, Marek Kluza, Krzysztof Czajkowski, Mirosław Wielgoś, Ewa Koc-Żórawska, Marcin Żórawski, Piotr Laudański

**Affiliations:** 1 II Department of Obstetrics and Gynecology, Medical University of Warsaw, 00-315 Warsaw, Poland; 2 Club 35. Polish Society of Gynecologists and Obstetricians, 53-125 Wrocław, Poland; 3 I Department of Obstetrics and Gynecology, Medical University of Warsaw, 02-015 Warsaw, Poland; 4 Department of Medical Informatics and Telemedicine, Medical University of Warsaw, 00-581 Warsaw, Poland; 5 Angelius Provita Hospital, 40-611 Katowice, Poland; 6 Division of Infertility and Reproductive Endocrinology, Department of Gynecology, Obstetrics and Gynecological Oncology, Poznan University of Medical Sciences, 61-701 Poznan, Poland; 7 Chair and Department of Laboratory Diagnostics, Poznan University of Medical Sciences, 61-701 Poznan, Poland; 8 Department of Obstetrics and Gynecology, Central Clinical Hospital of the Ministry of Interior, 02-507 Warsaw, Poland; 9 Center of Reproductive Health, Institute of Mother and Child in Warsaw, 01-211 Warsaw, Poland; 10 Department of Obstetrics and Gynecology, Institute of Mother and Child in Warsaw, 01-211 Warsaw, Poland; 11 Collegium Medicum, Jan Kochanowski University in Kielce, 25-369 Kielce, Poland; 12 Clinic of Obstetrics and Gynecology, Provincial Combined Hospital in Kielce, 25-736 Kielce, Poland; 13 Department of Gynecology and Obstetrics, Medical University of Lodz, 90-419 Lodz, Poland; 14 Department of Surgical Gynecology and Oncology, Medical University of Lodz, 90-419 Lodz, Poland; 15 Department of Fetal Medicine and Gynecology, Medical University of Lodz, 90-419 Lodz, Poland; 16 Clinic of Obstetrics and Gynecology in Przemysl, 37-700 Przemysl, Poland; 17 Department of Obstetrics and Gynecology, University of Rzeszow, 35-310 Rzeszow, Poland; 18 Department of Gynecology, Gynecology Oncology and Obstetrics, Institute of Medical Sciences, Medical College of Rzeszow University, 35-310 Rzeszow, Poland; 19 II Department of Nephrology and Hypertension with Dialysis Unit, Medical University of Bialystok, 15-089 Białystok, Poland; 20 Department of Clinical Medicine, Medical University of Bialystok, 15-089 Bialystok, Poland; 21 OVIklinika Infertility Center, 01-377 Warsaw, Poland

**Keywords:** endometriosis, ELISA, epithelial–mesenchymal transition, infertility, peritoneal fluid, plasma, ZEB1, ZEB2

## Abstract

Zinc finger E-box-binding homeobox 1 (ZEB1) and zinc finger E-box-binding homeobox 2 (ZEB2) are transcription factors that regulate epithelial–mesenchymal transformation (EMT). The aim of this study was to compare levels of ZEB1 and ZEB2 in the peritoneal fluid and plasma between patients with and without endometriosis in order to assess their utility in the diagnostic process. Plasma and peritoneal fluid samples were collected from 50 patients with and 48 without endometriosis during planned surgical procedures in eight clinical centers. Quantitative ZEB1 and ZEB2 levels analyses were performed using a double-antibody sandwich enzyme-linked immunosorbent assay (ELISA). No significant differences were observed in ZEB1 levels in any of the subanalyses nor any differences regarding ZEB2 levels between patients with and without endometriosis. Plasma ZEB2 levels were significantly higher among patients with infertility compared to fertile women (16.07 ± 12.70 ng/L vs. 12.07 ± 11.92 ng/L; *p* < 0.04). Both ZEB1 and ZEB2 do not seem to have a significant value in the initial diagnosis of endometriosis as a single marker. The differences in ZEB2 plasma levels between patients with and without infertility indicate the possibility of EMT dysregulation in the pathogenesis of adverse fertility outcomes.

## 1. Introduction

Endometriosis is a common, estrogen-dependent inflammatory disease characterized by the presence of endometrial tissue outside the uterus [1,2]. Based on Sampson’s most popular theory, it is the result of retrograde menstruation [3]. Endometriosis, with its high prevalence worldwide, affects 6–10% of reproductive-age women and even half of women with fertility problems [2,4,5]. Multiple immunological and neoangiogenic factors are examined in order to better understand endometriosis pathogenesis and establish causative therapeutic agents in future [4,6,7,8]. Unfortunately, diagnosis of endometriosis is still associated with challenges. Due to vague symptoms of the disease and invasive character of surgery needed for a definitive diagnosis in some cases, the diagnostic delay may exceed seven years [9,10].

Zinc finger E-box-binding homeobox 1 (ZEB1) and zinc finger E-box-binding homeobox 2 (ZEB2) are members of zinc-finger E-box-binding transcription factors that regulate cellular transformation. Both proteins participate in cancer development and progression by repressing adhesion molecules such as E-cadherin—a major adhesion molecule of epithelial cells [11,12]. E-cadherin is organized in cadherin–catenin transmembrane complexes localized in cell adherence junctions and plays a role in stabilization and homeostasis of epithelial cells [13]. Downregulation of E-cadherin is the EMT-inducing factor [14]. ZEB1 and ZEB2 are known as E-cadherin-repressing transcription factors [13,14]. For this reason, these molecules can have a critical role in the epithelial–mesenchymal transition (EMT) due to both E-cadherin repression and through transforming growth factor beta (TGF-β) or bone morphogenetic proteins (BMPs) signaling, the nuclear factor kappa-light-chain-enhancer of activated B cells NF-κB, and the Notch signaling pathways [15].

The role of EMT has not only been demonstrated in the pathogenesis of oncological diseases, such as non-small cell lung cancer, pancreatic cancer, colorectal cancer or gastric cancer, but also in endometriosis [15,16,17,18]. Properly functioning mesothelial barrier prevents stromal cells from endometrial cells’ invasion and the formation of extrauterine endometrial lesions [19]. It was observed in genome-wide association studies that endometriosis is linked with genes (WNT4, CDC42, ID4, VEZT) actively participating in the regulation of cytoskeleton and mesothelial barrier function [19]. Altered expression of mentioned genes affects the EMT process [19]. Induction of EMT contributes to a change in cell phenotype, enabling cell invasion and migration, which are both elements of endometriosis pathogenesis [20,21,22,23].

It was proved that there is a coexistence of EMT and higher expression of proteins from ZEB family [11,24,25,26]. There is also an example of higher ZEB1 expression in tissues altered by endometriosis [27]. Knowledge about ZEB2 involvement in endometriosis pathomechanism is much less advanced. Based on the accessible literature, higher ZEB2 expression has been observed in endometriotic tissue of patients with endometriosis [28]. There are no such studies regarding peritoneal fluid or plasma. Analysis of other specimen types can be useful, as not only endometriotic tissue but also the peritoneal fluid is a recognized biological material that can be investigated in order to better understand the environment and the pathogenesis of endometriosis [29,30,31]. This is not to mention the plasma, which is a much less invasively obtainable specimen.

The aim of this study was to compare levels of ZEB1 and ZEB2 in the peritoneal fluid and plasma between patients with and without endometriosis in order to assess their utility in the endometriosis diagnostic process.

## 2. Materials and Methods

This was a multicenter, cross-sectional study. Plasma and peritoneal fluid samples were collected from 50 patients with (study group) and 48 without (control group) endometriosis during planned surgical procedures in eight Polish clinical centers between 2018 and 2019: I Department of Obstetrics and Gynecology, Medical University of Warsaw; Angelius Provita Hospital in Katowice; Department of Gynecology, Division of Infertility and Reproductive Endocrinology, Obstetrics and Gynecological Oncology at Poznan University of Medical Sciences; Department of Obstetrics and Gynecology, Central Clinical Hospital of the Ministry of Interior in Warsaw; Clinic of Obstetrics and Gynecology, Provincial Combined Hospital in Kielce; Department of Surgical Gynecology and Oncology, Medical University of Lodz; Department of Gynecology and Obstetrics, Provincial Hospital in Przemysl; Department of Gynecology, Gynecology Oncology and Obstetrics, Institute of Medical Sciences, Medical College of Rzeszow University.

The study group consisted of patients between 18 and 40 years old who qualified for planned laparoscopic surgeries due to one or more non-malignant conditions: infertility, chronic pelvic pain syndrome, ovarian cysts, suspicion of endometriosis. Exclusion criteria were: irregular menstruations, hormonal treatment within three months before the surgery, pelvic inflammatory disease, uterine fibroids, polycystic ovary syndrome, autoimmune comorbidities, malignancies, and any previous history of surgical treatment. Each patient was evaluated on the basis of the revised American Fertility Society (AFS) classification of endometriosis, together with histology examination of collected specimens [32]. All patients completed the World Endometriosis Research Foundation (WERF) clinical questionnaire [33].

Patients without the confirmation of a visible endometriosis during laparoscopy were recruited to control group. Based on inspection during laparoscopy, patients with endometriosis were allocated to the adequate endometriosis stage subgroup (I–IV). Additionally, prior to the surgery, blood samples were collected in ethylenediaminetetraacetic acid (EDTA) 10 mL tubes (Sarstedt) in order to obtain specimen for plasma evaluation. Peritoneal fluid was collected via Veress needle aspiration under direct visual inspection in the beginning of the laparoscopy in order to avoid contamination with blood. The procedure was every time performed in accordance with the Endometriosis Phenome and Biobanking Harmonisation Project standard operating procedures [34]. Material collection did not have any impact on medical management of patients and was performed in the manner of the Declaration of Helsinki. Aspirated peritoneal fluid was spun in all centers at 1000× *g* for 10 min at 4 °C. The supernatant was transferred to a fresh 10 mL tube (Sarstedt). The same types of tubes were used for blood and peritoneal fluid in all centers. The time lapse between sample collection (both peritoneal fluid and plasma) and processing was less than 45 min. All centers centrifuged blood samples at 2500× *g* for 10 min at 4 °C. Specimen samples were stored at −80 °C.

Double-antibody sandwich enzyme-linked immunosorbent assay (ELISA) was performed in order to assess levels of both ZEB1 and ZEB2 in collected plasma and peritoneal fluid samples. ELISA is a quantitative method which has been used for detection and quantification of specific substances [35]. It has been performed in order to detect ZEB1 and ZEB2 in human biological samples. Human ZEB1 kits (SunRedBio, Shanghai, China, Catalogue number SRB-T-88280) were used with the sensitivity of 0.186 ng/L and assay range 0.2–60 ng/L. Human ZEB2 kits (SunRedBio, Shanghai, China, Catalogue number 201-12-5968) were used with the sensitivity of 0.135 ng/L and assay range 0.15–32 ng/L. Study protocol was approved by the Institutional Review Board at the Medical University of Warsaw (approval number AKBE/132/2020).

Outliers were detected and then excluded using classic statistical domain based on interquartile range. After exclusion of the outlier results, 75 samples of plasma (42 from patients with and 33 from patients without endometriosis) and 76 samples of the peritoneal fluid (44 from patients with and 32 from patients without endometriosis) were included in the final analyses for ZEB1. For ZEB2, the numbers were as follows: 88 samples of plasma (50 from patients with and 38 from patients without endometriosis) and 84 samples of peritoneal fluid (47 from patients with and 37 from patients without endometriosis).

Statistical analysis was performed with SAS v. 9.4 (SAS Institute, Cary, NC, USA) and Statistica v. 13.3 (StatSoft Inc., Crocow, Poland) software. The groups were compared by Chi-square test for categorical variables. Mann–Whitney U-test and t-Student’s test were performed for continuous variables depending on the distribution of variables after testing for normal distribution using Shapiro–Wilk test. In case of multiple variables measurement Kruskal–Wallis ANOVA analysis was used. The level of statistical significance was set at *p* < 0.05.

## 3. Results

Table 1 presents ZEB1 and ZEB2 plasma concentrations depending on endometriosis and infertility presence as well as menstrual cycle phase. No significant differences were observed in ZEB1 levels. There were significant differences observed in ZEB2 levels between infertile and fertile patients (16.07 ± 12.70 ng/L vs. 12.07 ± 11.92 ng/L; *p* < 0.04). The difference was still observed in terms of primary infertility (17.27 ± 12.14 ng/L vs. 12.14 ± 11.71 ng/L; *p* < 0.04), but not in terms of the secondary one.

Table 2 presents ZEB1 and ZEB2 plasma levels in patients with confirmed endometriosis depending on its stage—no significant differences were observed.

ZEB1 and ZEB2 concentrations in the peritoneal fluid are presented in Table 3. No significant differences depending on endometriosis presence, infertility, and menstrual cycle phase were observed.

Table 4 presents ZEB1 and ZEB2 concentrations in the peritoneal fluid in patients with confirmed endometriosis depending on its stage—no significant differences were observed.

## 4. Discussion

Endometriosis as a disease creates difficulties at all stages of clinical practice, starting from diagnosis and ending with treatment. Currently, two approaches to diagnosis are most commonly used—presumptive diagnosis or definite surgical diagnosis [36]. The first one consists of combined patient’s signs and symptoms, physical examination and most commonly transvaginal ultrasonography [37]. Components of nonsurgical diagnostic process may include: physical examination findings of rectovaginal lesions confirmed in ultrasound, ultrasonographic finding of ovarian endometrioma, evaluation of posterior cervical fornix with optional biopsy of rectovaginal lesions (if applicable), and cystoscopy [37]. This approach is relatively safe and, with a consecutive, empirical use of hormonal contraception or progestogens, can lead to relief of symptoms at a low cost [38]. The surgical diagnosis performed usually during laparoscopy enables identification of asymptomatic endometriosis, which is common and especially important in the context of infertility and helps in a more accurate assessment of disease severity [39]. Surgical diagnosis includes visual inspection based on the revised American Fertility Society (AFS) classification and/or histology of suspected endometrial lesions [32]. However, as with all invasive interventions, it can lead to surgical complications and is much more expensive for the medical system [39]. Investigation of novel potential endometriosis biomarkers could lead to quicker identification of patients requiring treatment and shortening the diagnostic delay, which currently lasts up to several years [9]. As there are high potential benefits of having such markers, numerous studies analyzed the subject searching for a highly specific and sensitive diagnostic tool [40,41]. Unfortunately, in the results of our study, we did not observe any association between plasma or peritoneal fluid zinc finger E-box-binding homeobox 1 and 2 levels and the incidence of endometriosis.

On the contrary, several available studies indicate that there is a link between ZEB expression in the endometrial tissue, EMT dysregulation and endometriosis pathogenesis. Instability of the mesothelial barrier caused by EMT induction enables cell invasion, which is necessary for development of endometrial peritoneal lesions [19,22,23]. ZEB proteins are transcription factors contributing to EMT dysregulation by E-cadherin repression. One of the first reports exploring ZEB1 expression in the pathomechanism of endometriosis was published in 2017 by Furuya et al. [27]. The authors observed that ZEB1 expression was mostly associated with invasive lesions. Among the examined samples, ZEB1 expression in epithelia was detected in 83.3% cases of adenomyosis, 80% cases of deep infiltrating endometriosis, and 16.7% cases of ovarian endometrioma. No ZEB1 staining was observed in luminal nor glandular epithelial cells of eutopic normal endometrial tissue collected from endometriosis patients [27]. In a systematic review by Konrad et al., it was presented that, in all studies identified in the literature, the expression of ZEB1 was significantly higher in the ectopic than in the eutopic endometrial tissue [42]. What is more, Wu et al. observed that following ZEB1 downregulation it was possible to suppress EMT in Ishikawa cell line derived from endometrial adenocarcinoma, which suggests a new potential therapeutic target [43]. In a study by Wang et al. authors examined expression levels of ZEB2 and other EMT-related genes in human specimen collected from ectopic endometrial tissue. A significant ZEB2 overexpression in endometriosis tissue samples was identified compared to levels detected in the normal endometrium [44]. The reason why ZEB expression in tissues widely reported in the literature was not reflected in fluids levels in our study could be that the target of ZEB proteins are E-boxes of epithelial gene promoter regions located inside epithelial cells [12,45,46].

We have also analyzed the association between ZEB levels and the history of infertility. The epithelial–mesenchymal transition is one of crucial processes during embryogenesis and tissue differentiation [47]. ZEB proteins participate in the activation of the EMT mechanism by repressing epithelial and promoting mesenchymal markers expression [48]. ZEB1 directly represses the expression of Crumbs3 and mucin-1, while ZEB2 directly downregulates tight junction proteins claudin-4, ZO-3 and desmosome protein plakophilin-2, and induces vimentin expression [49,50,51,52,53]. During type 1 EMT, which enables embryogenesis, mesenchymal cells are able to produce secondary epithelium due to the mesenchymal–epithelial transition (MET) process [20]. Cela et al. investigated serum and intrafollicular concentrations of EMT markers in patients with endometriosis and infertility [54]. The authors observed that epithelial markers were significantly decreased while mesenchymal markers were significantly increased in endometriosis patients compared to controls. At the same time, the better IVF outcome defined by the higher number of MII oocytes and good quality embryos was positively associated with E-cadherin (epithelial marker) expression. This stands in line with the results of our study, as patients with infertility and primary infertility presented significantly higher plasma ZEB2 levels, which downregulates E-cadherin [55]. Our results indicate that infertility can be associated with EMT dysregulation. In a study by Ishida et al., the authors evaluated endometrial samples from 66 infertile patients [56]. One of the analyzed characteristics was the presence of active EMT process defined as a loss of E-cadherin expression and/or detected N-cadherin expression. It was observed that chronic endometritis was more frequently prevalent in patients with EMT-positive samples than in specimens without detected EMT (74% vs. 34%, *p* = 0.0015). In the same study, the authors analyzed specimen collected from infertile patients with and without endometriosis. No significant difference regarding the occurrence of endometriosis was observed between EMT-positive (32%) and EMT-negative (17%) endometrial samples (*p* = 0.25) [56].

Study limitations of our protocol include a relatively small sample size; however, it is still larger than in numerous previously published articles on ZEB expression in women with endometriosis, as our patients’ enrollment was performed in a multicenter study at eight clinical sites [20,21,36,37]. The strength of the study involves strict exclusion criteria and simultaneous analysis of an easily accessible specimen, which is plasma, and a specimen obtained during an invasive procedure—peritoneal fluid.

## 5. Conclusions

Both ZEB1 and ZEB2 do not seem to have a significant value in the initial diagnosis of endometriosis as a single marker. Evaluation of the levels of ZEB1 and ZEB2 in peritoneal fluid performed after surgery does not seem to have significant value. The possible differences in ZEB2 plasma levels between patients with and without infertility indicate the possibility of EMT dysregulation in the pathogenesis of adverse fertility outcome, but further studies on a wider group of patients are required.

## Figures and Tables

**Table 1:** **Zinc finger E-box-binding homeobox 1** (ZEB1) and Zinc finger E-box-binding homeobox 2 (ZEB2) plasma concentrations.

**ZEB1**			
**Feature**	**Present**	**Absent**	***p***
Endometriosis [ng/L; ±SD]	22.18 ± 16.43	20.21 ± 14.24	0.78
Infertility [ng/L; ±SD]	23.32 ± 16.26	18.89 ± 14.27	0.19
Primary infertility [ng/L; ±SD]	23.82 ± 17.55	19.73 ± 13.92	0.62
Secondary infertility [ng/L; ±SD]	22.12 ± 13.22	21.16 ± 15.92	0.28
	**First**	**Second**	
Menstrual cycle phase [ng/L; ±SD]	21.26 ± 16.03	21.42 ± 14.57	0.97
**ZEB2**			
**Feature**	**Present**	**Absent**	***p***
Endometriosis [ng/L; ±SD]	15.22 ± 12.91	13.09 ± 11.89	0.53
Infertility [ng/L; ±SD]	**16.07** **±** **12.70**	**12.07** **±** **11.92**	**0.04**
Primary infertility [ng/L; ±SD]	**17.27** **±** **12.14**	**12.14** **±** **11.71**	**0.04**
Secondary infertility [ng/L; ±SD]	12.35 ± 11.57	14.60 ± 12.64	0.99
	**First**	**Second**	
Menstrual cycle phase [ng/L; ±SD]	14.02 ± 12.69	14.17 ± 11.90	0.88

**Table 2:** Zinc finger E-box-binding homeobox 1 (ZEB1) and Zinc finger E-box-binding homeobox 2 (ZEB2) plasma concentrations depending on the stage of endometriosis.

Endometriosis Stage.	I	II	III	IV	*p*
ZEB1 [ng/L; ±SD]	27.77 ± 18.92	20.72 ± 21.16	20.65 ± 17.10	20.03 ± 8.76	0.63
ZEB2 [ng/L; ±SD]	17.67 ± 12.57	17.46 ± 16.80	13.62 ± 12.93	9.48 ± 7.07	0.62

**Table 3:** Zinc finger E-box-binding homeobox 1 (ZEB1) and Zinc finger E-box-binding homeobox 2 (ZEB2) concentrations in the peritoneal fluid.

**ZEB1**			
**Feature**	**Present**	**Absent**	***p***
Endometriosis [ng/L; ±SD]	22.69 ± 18.79	16.69 ± 11.76	0.50
Infertility [ng/L; ±SD]	21.89 ± 17.62	17.36 ± 13.99	0.48
Primary infertility [ng/L; ±SD]	23.44 ± 18.93	16.88 ± 12.79	0.94
Secondary infertility [ng/L; ±SD]	15.32 ± 8.26	20.91 ± 17.12	0.70
	**First**	**Second**	
Menstrual cycle phase [ng/L; ±SD]	19.00 ± 15.37	22.26 ± 18.20	0.47
**ZEB2**			
**Feature**	**Present**	**Absent**	***p***
Endometriosis [ng/L; ±SD]	17.91 ± 26.53	14.32 ± 11.21	0.81
Infertility [ng/L; ±SD]	16.61 ± 20.09	14.92 ± 18.78	0.11
Primary infertility [ng/L; ±SD]	18.23 ± 21.78	13.79 ± 17.03	0.07
Secondary infertility [ng/L; ±SD]	9.41 ± 5.93	16.69 ± 20.36	0.66
	**First**	**Second**	
Menstrual cycle phase [ng/L; ±SD]	12.52 ± 11.53	22.67 ± 28.64	0.10

**Table 4:** Zinc finger E-box-binding homeobox 1 (ZEB1) and Zinc finger E-box-binding homeobox 2 (ZEB2) peritoneal concentrations depending on the stage of endometriosis.

Endometriosis Stage	I	II	III	IV	*p*
ZEB1 [ng/L; ±SD]	22.51 ± 17.82	27.18 ± 24.20	22.36 ± 18.62	21.52 ± 19.25	0.99
ZEB2 [ng/L; ±SD]	15.82 ± 11.46	13.40 ± 12.90	14.28 ± 12.32	13.98 ± 9.13	0.87

## Data Availability

The data presented in this study are available on request from the corresponding author. The data are not publicly available due to privacy.
